# Two Novel Adenovirus Vectors Mediated Differential Antibody Responses via Interferon-α and Natural Killer Cells

**DOI:** 10.1128/spectrum.00880-23

**Published:** 2023-06-22

**Authors:** Peng Zou, Panli Zhang, Qitao Deng, Cong Wang, Shengxue Luo, Ling Zhang, Chengyao Li, Tingting Li

**Affiliations:** a Department of Transfusion Medicine, School of Laboratory Medicine and Biotechnology, Southern Medical University, Guangzhou, China; b Department of Pediatrics, Shenzhen Hospital, Southern Medical University, Shenzhen, China; Technion—Israel Institute of Technology

**Keywords:** novel adenovirus vector, antibody response, interferon-α, NK cells, adenoviruses, antibody function, interferons

## Abstract

Recombinant adenovirus vectors have been widely used in vaccine development. To overcome the preexisting immunity of human adenovirus type 5 (Ad5) in populations, a range of chimpanzee or rare human adenovirus vectors have been generated. However, these novel adenovirus vectors mediate the diverse immune responses in the hosts. In this study, we explored the immune mechanism of differential antibody responses to SARS-CoV-2 S protein in mice immunized by our previously developed two novel simian adenovirus type 23 (Sad23L) and human adenovirus type 49 (Ad49L), and Ad5 vectored COVID-19 vaccines. Sad23L-nCoV-S and Ad5-nCoV-S vaccines induced the low level of interferon-α (IFN-α) and the high level of antigen-specific antibody responses in wild-type and IFN-α/β receptor defective (IFNAR^−/−^) C57 mice, while Ad49L-nCoV-S vaccine induced the high IFN-α and low antibody responses in C57 mice but the high antibody response in IFNAR^−/−^ mice. In addition, the high antibody response was detected in natural killer (NK) cells-blocked but the low in follicular helper T (T_FH_) cells -blocked C57 mice immunized with Ad49L-nCoV-S vaccine. These results showed that Ad49L vectored vaccine stimulated IFN-α secretion to activate NK cells, and then reduced the number of T_FH_ cells, generation center (GC) B cells and plasma cells, and subsequently reduced antigen-specific antibody production. The different novel adenovirus vectors could be selected for vaccine development according to the need for either humoral or cellular or both immune protections against a particular disease.

**IMPORTANCE** Novel adenovirus vectors are an important antigen delivery platform for vaccine development. Understanding the immune diversity between different adenoviral vectors is critical to design the proper vaccine against an aim disease. In this study, we described the immune mechanism of Sad23L and Ad49L vectored vaccines for raising the equally high specific T cell response but the different level of specific antibody responses in mice. We found that Ad49L-vectored vaccine initiated the high IFN-α and activated NK cells to inhibit antibody response via downregulating the number of CD4^+^ T_FH_ cells leading to the decline of GC B cells and plasma cells.

## INTRODUCTION

Adenovirus (Ad) vectors have proven to be very effective to mediate antigen-specific T and B cell responses. The recombinant human adenovirus type 5 (Ad5) vector has been widely used for vaccine study in the laboratory. Due to the widespread preexisting immunity to Ad5 in the humans, the immune efficacy of Ad5 vectored vaccines is restricted (1, 2). In order to overcome the preexisting immunity, a range of rare serotype adenovirus vectors have been developed (3–6). However, some novel Ad vectors constructed from low-seroprevalence adenoviruses have shown the various status of immune responses *in vivo* (7–9). The reasons for this difference between Ad vectors are poorly understood, yet it is critical for using the proper Ad vectors for the development of candidate vaccines based upon these adenoviral characteristics.

In our previous studies, two novel adenoviral vectors, Sad23L and Ad49L, derived from a simian adenovirus type 23 (Sad23) or a human adenovirus type 49 (Ad49), were constructed and used for development of Zika and novel coronavirus disease 2019 (COVID-19) vaccines (10–12). Both Sad23L and Ad49L vectored vaccines raised the strongly specific T cell response, while Sad23L vectored vaccine induced the high but Ad49L vectored vaccine induced the low level of specific antibody responses (11). Previous studies indicated that type I interferon (IFN-I) had an inverse role for production of antigen-specific antibodies from a chimpanzee adenovirus or lymphocytic choriomeningitis virus (LCMV) infection (9, 13). The adenovirus serotypes differ in infectivity, cellular receptor, intracellular trafficking route, and genome CpG content, but these factors have not been conclusively shown to be directly responsible for differentiating immunogenicity (14–17).

In this study, we sought the role of IFN-α on transgene antigen-specific antibody responses induced by these two novel adenovirus vectored COVID-19 vaccines (Sad23L-nCoV-S and Ad49L-nCoV-S) in comparison with Ad5-nCoV-S vaccine and found that the early status of IFN-α response could be a key factor for triggering activation of natural killer (NK) cells, and subsequently inhibiting production of antigen-specific antibody.

## RESULTS

### Two novel adenovirus vectored COVID-19 vaccines induced the differential immune responses in C57 mice.

**FIG 1:**
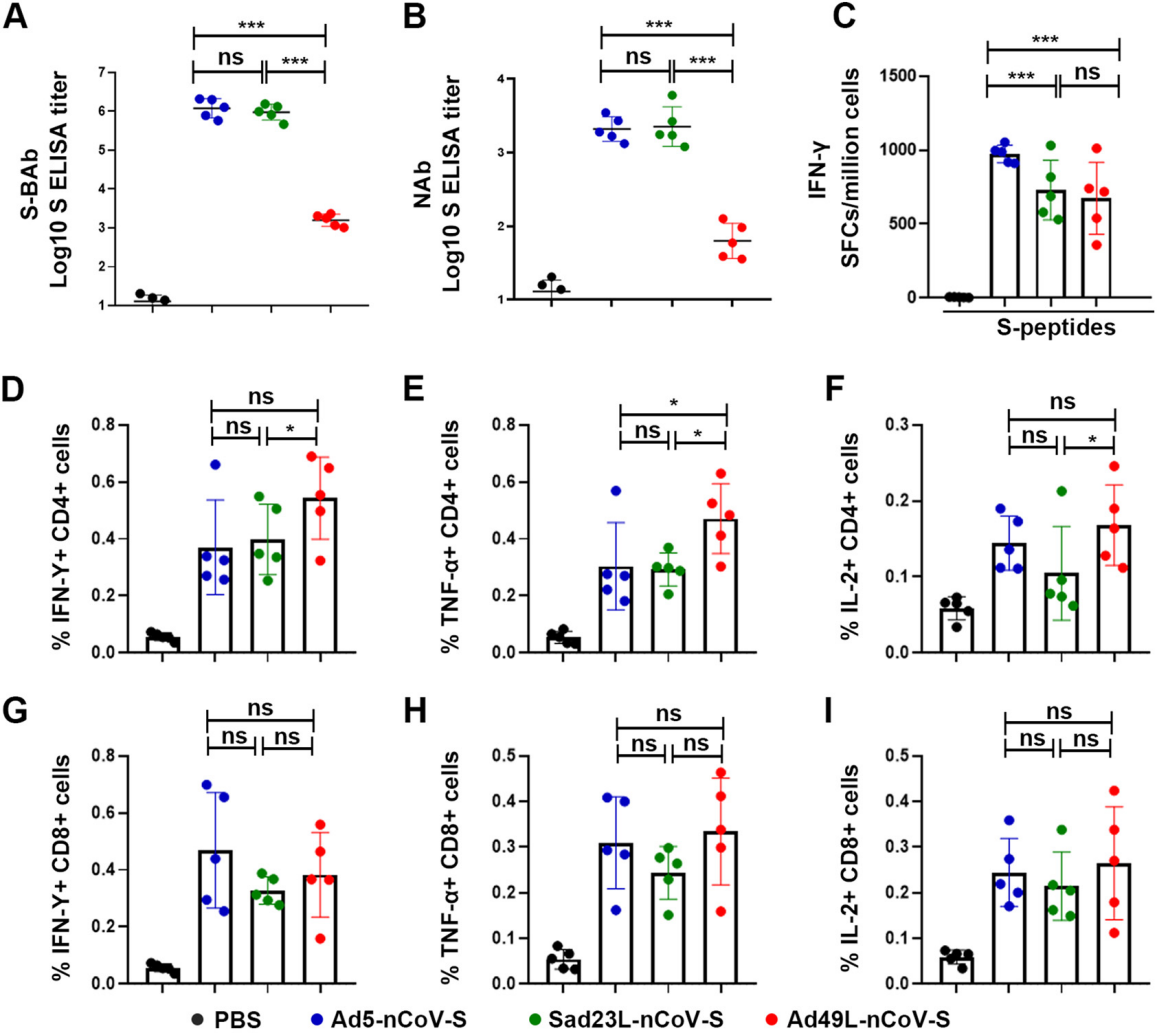
Specific antibody and T-cell response of C57 mice inoculated with Sad23L-nCoV-S, Ad49L-nCoV-S, or Ad5-nCoV-S. C57 mice (*n* = 5/group) were immunized by a dose of 10^9^ PFU Sad23L-nCoV-S, Ad49L-nCoV-S, or Ad5-nCoV-S. Mice sera and splenocytes were collected for measurement of antibody and T-cell responses 4 weeks postimmunization. (A) S-BAb titers weekly were obtained by ELISA. (B) NAb titers were obtained by pVNT. (C) IFN-γ secreting T-cell response (SFCs/million cells) of splenocytes to S peptides from Sad23L-nCoV-S, Ad49L-nCoV-S, or Ad5-nCoV-S immunized mice was measured by ELISpot, respectively. (D-I) Frequency of IFN-γ, TNF-α or IL-2 expressing CD4^+^ and CD8^+^ T-cell response to S peptides determined by ICS. *P* values are analyzed by one-way ANOVA with 2-fold Bonferroni adjustment. Statistically significant differences are shown with asterisks (*, *P* < 0.05; **, *P* < 0.01 and ***, *P* < 0.001).

A single dose of 10^9^ PFU Sad23L-nCoV-S or Ad49L-nCoV-S in comparison with Ad5-nCoV-S vaccine was intramuscularly injected to C57BL/6 (C57) mice (*n* = 5/group), respectively, and an equal volume of PBS was used as naive control group (*n* = 5). Serum sample was collected in a month postimmunization. The SARS-CoV-2 S protein binding antibody (S-BAb) and neutralizing antibody (NAb) titers were measured by the enzyme linked immunosorbent assay (ELISA) and pseudovirus neutralization test (pVNT), and the specific T-cell response was detected by the enzyme linked immunospot assay (ELISpot) and flow cytometry, respectively. The titers of 9.53 × 10^5^ and 1.20 × 10^6^ for S-BAb and 2.25 × 10^3^ and 2.04 × 10^3^ for NAb were detected from Sad23L-nCoV-S or Ad5-nCoV-S immunized mice, respectively (Fig. 1A and B), which were significantly higher than those (BAb 1.6 × 10^3^ or NAb 6.31 × 10^1^) from Ad49L-nCoV-S immunized mice (*P* < 0.001).

Interestingly, Ad5-nCoV-S elicited stronger specific IFN-γ secreting T-cell response to S peptides (973.60 SFCs/10^6^ cells) than Sad23L-nCoV-S or Ad49L-nCoV-S (729.52 or 673.36 SFCs/10^6^ cells) (Fig. 1C) (*P* < 0.001), while Ad49L-nCoV-S stimulated higher IFN-γ^+^, TNF-α^+^ and IL-2^+^ CD4^+^ T cell responses than Sad23L-nCoV-S or Ad5-nCoV-S (Fig. 1D to F) (*P* < 0.05). Similar levels of these cytokine positive CD8^+^ T cell responses were observed by flow cytometry among Sad23L-nCoV-S, Ad5-nCoV-S and Ad49L-nCoV-S vaccinated mice (Fig. 1G-I).

Overall, Sad23L and Ad5 vectored vaccines elicit both higher specific antibody and T cell responses, while Ad49L vectored vaccine induced lower antibody but higher T-cell responses in C57 mice.

### Characterization of immune responses in three adenovirus vectored COVID-19 vaccines inoculated IFNAR^−/−^ mice.

**FIG 2:**
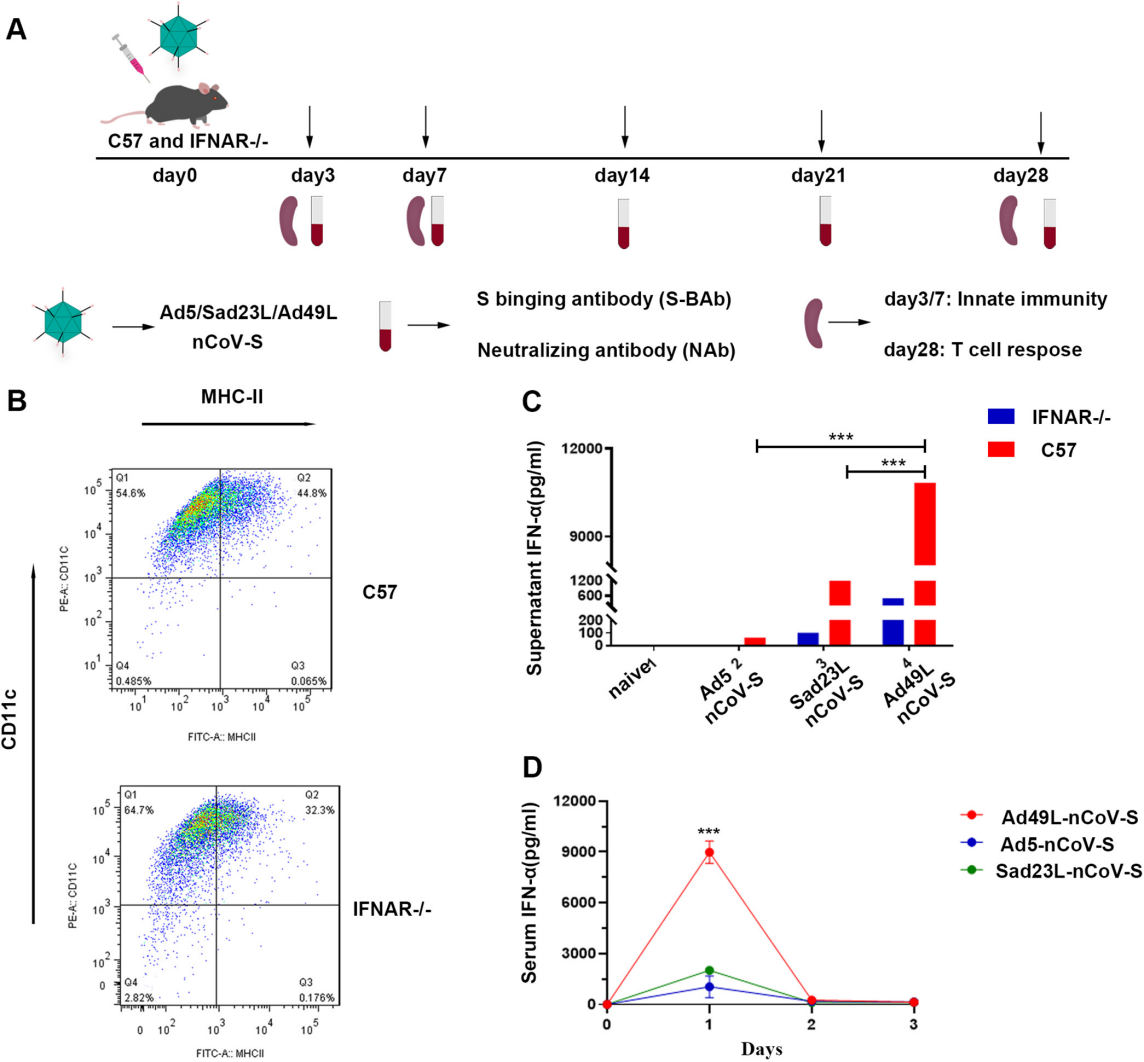
IFN-I levels of wild-type C57 and IFNAR^−/−^ C57 mice induced by Ad5-nCoV-S, Sad23L-nCoV-S, or Ad49L-nCoV-S. Dendritic cells (DC) were obtained from C57 and IFNAR^−/−^ mice infected with Ad5-nCoV-S, Sad23L-nCoV-S, or Ad49L-nCoV-S. The serum of C57 and IFNAR^−/−^ mice was collected 3 days after immunization. IFN-α in DC supernatant and serum samples were detected by mouse IFN-α ELISA kit. (A) C57 mice (*n* = 5/group) were immunized by a dose of 10^9^ PFU Ad5-nCoV-S, Sad23L-nCoV-S, or Ad49L-nCoV-S. (B) Measuring of IFN-α in DC culture supernatants from C57 and IFNAR^−/−^ mice. (C) IFN-α in supernatants of DC cultures from C57 and IFNAR^−/−^ mice, respectively. (D) IFN-α in sera from C57 and IFNAR^−/−^ mice during 3 days after immunization. Statistically significant differences are shown with asterisks (*, *P* < 0.05; **, *P* < 0.01 and ***, *P* < 0.001).

**FIG 3:**
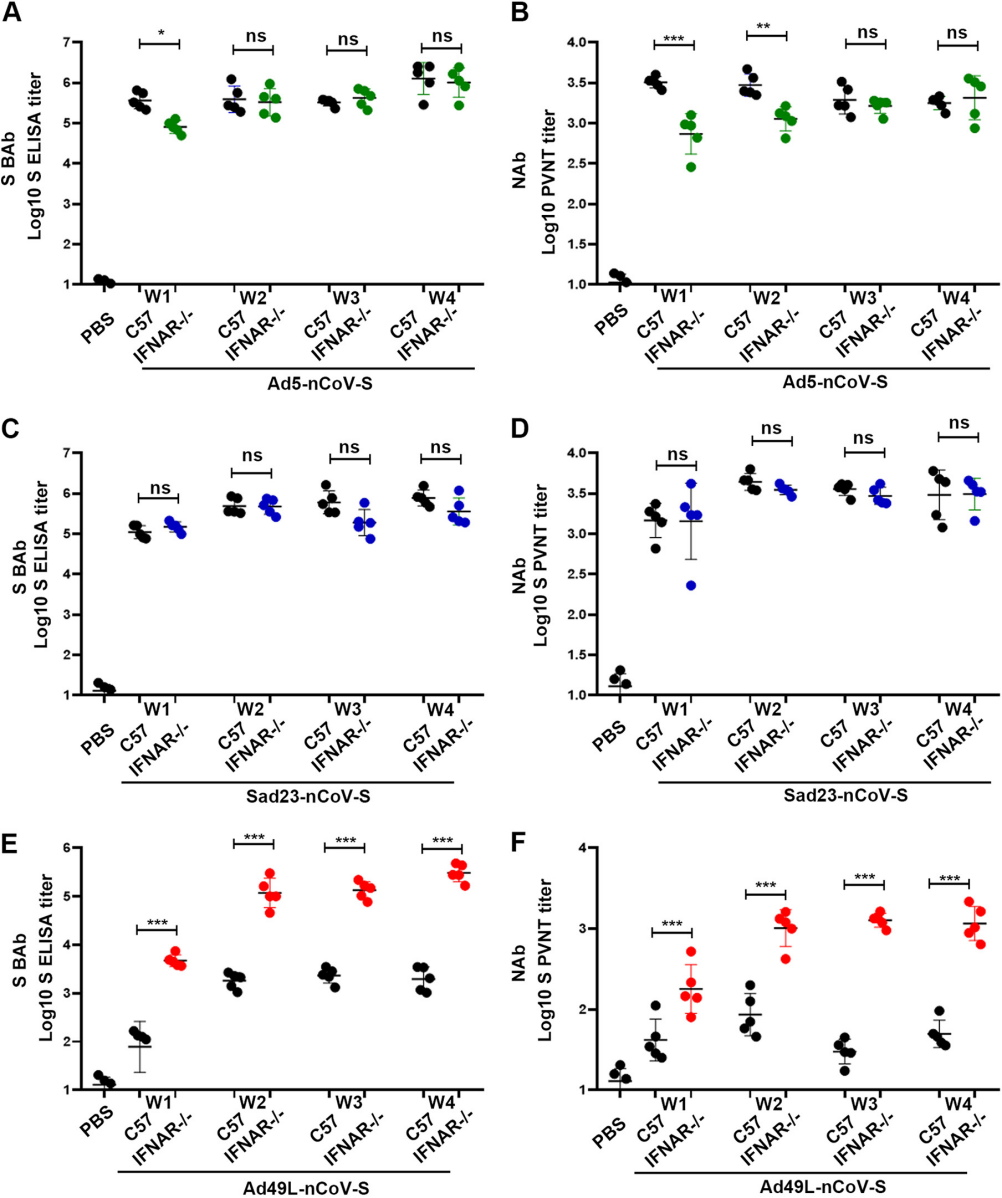
Specific antibody response of wild-type C57 and IFNAR^−/−^ C57 mice immunized with Ad5-nCoV-S, Sad23L-nCoV-S, or Ad49L-nCoV-S. C57 and IFNAR^−/−^ mice (*n* = 5/group) were immunized by a dose of 10^9^ PFU Ad5-nCoV-S, Sad23L-nCoV-S, or Ad49L-nCoV-S. (A-B) S-BAb and NAb titers of C57and IFNAR^−/−^ mice immunized with Ad5-nCoV-S. (C-D) S-BAb and S-NAb titers of C57 and IFNAR^−/−^ mice immunized with Sad23L-nCoV-S. (E-F) S-BAb and S-NAb titers of C57 and IFNAR^−/−^ mice immunized with Ad49L-nCoV-S. Statistically significant differences are shown with asterisks (*, *P* < 0.05; **, *P* < 0.01 and ***, *P* < 0.001).

By comparing with C57 mice, IFNAR^−/−^ mice were immunized with a single dose of 10^9^ PFU Sad23L-nCov-S, Ad49L-nCov-S, or Ad5-nCov-S vaccine, respectively, and then examined for IFN-α, antibody, and T cell responses at various time points during 4 weeks postinoculation (Fig. 2A). Dendritic cells (DCs) were isolated from bone marrow cells of C57 and IFNAR^−/−^ mice and stimulated with granulocyte-macrophage colony-stimulating factor (GM-CSF) and interleukin (IL)-4 (Fig. 2B). IFN-α was measured in the supernatants of DC cultures (Fig. 2C) and serum samples from mice during 3 days postinoculation (Fig. 2D). The levels of IFN-α in supernatant of DC cultures and serum from Ad49L-nCoV-S immunized mice were obviously higher than Sad23L-nCoV-S or Ad5-nCoV-S immunized mice (Fig. 2C and D) (*P* < 0.001). Both S-BAb and NAb titers had insignificant difference between C57 and IFNAR^−/−^ mice vaccinated with Ad5-nCov-S (Fig. 3A and B) or Sad23L-nCoV-S (Fig. 3C and D) (*P* > 0.05), while these two kinds of antibody titers varied significantly between Ad49L-nCoV-S immunized C57 and IFNAR^−/−^ mice (Fig. 3E and F) (*P* < 0.001). Intriguingly, Ad49L-nCov-S vaccinated IFNAR^−/−^ mice had a high level of antibody response similar to Ad5-nCov-S or Sad23L-nCoV-S immunized IFNAR^−/−^ and C57 mice, which was much higher than Ad49L-nCoV-S vaccinated C57 mice (Fig. 3) (*P* < 0.001). These observations suggested that Ad49L vectored vaccine increased IFN-α expression and played an important role for negatively correlating with antigen-specific antibody response.

**FIG 4:**
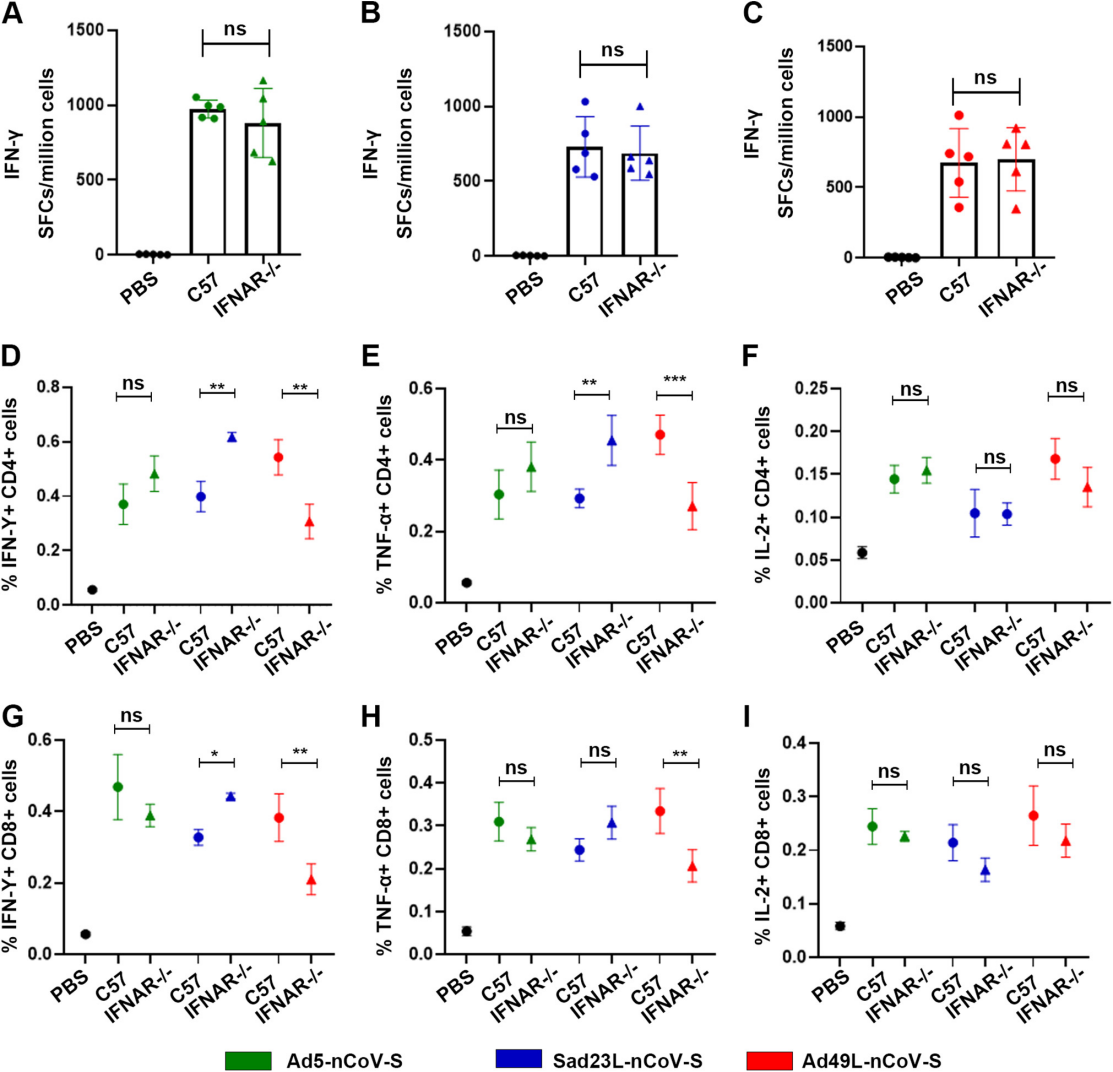
T cell response of C57 and IFNAR^−/−^ mice immunized with Ad5-nCoV-S, Sad23L-nCoV-S, or Ad49L-nCoV-S. C57 and IFNAR^−/−^ mice (*n* = 5/group) were immunized by a dose of 10^9^ PFU Ad5-nCoV-S, Sad23L-nCoV-S, or Ad49L-nCoV-S. (A-C) IFN-γ secreting T-cell response of splenocytes to S peptides from Ad5-nCoV-S, Sad23L-nCoV-S, or Ad49L-nCoV-S immunized C57and IFNAR^−/−^ mice was measured by ELISpot. (D-I) Frequency of IFN-γ, TNF-α or IL-2expressing CD4^+^ and CD8^+^ T-cell response to S peptides determined by ICS. Statistically significant differences are shown with asterisks (*, *P* < 0.05; **, *P* < 0.01 and ***, *P* < 0.001).

The antigen-specific T-cell responses were detected in both C57 and IFNAR^−/−^ mice immunized by these three vaccines of Sad23L-nCoV-S, Ad49L-nCoV-S and Ad5-nCoV-S (Fig. 4), of which IFN-γ secreting T cell responses were in a similar high level (650-970 SFCs/10^6^ cells) between wild-type and IFNAR^−/−^ mice (*P* > 0.05; Fig. 4A-C), while IFN-γ^+^, TNF-α^+^ and CD4^+^/CD8^+^ T cell responses were higher in Ad49L-nCoV-S immunized C57 mice but lower in IFNAR^−/−^ mice (*P* < 0.01 or 0.001; Fig. 4D-I). Comparably, Sad23L-nCoV-S presented a reverse pattern to Ad49L-nCoV-S for lower IFN-γ^+^ and TNF-α^+^ CD4^+^ and CD8^+^ T cell responses in C57 but higher in IFNAR^−/−^ mice (*P* < 0.05). Ad5-nCoV-S induced CD4^+^ or CD8^+^ T cell responses varied insignificantly between the two types of mice (*P* > 0.05; Fig. 4D-I). These data suggested that the two novel adenovirus vectors Sad23L and Ad49L oppositely mediated CD4^+^ or CD8^+^ T cell responses through involvement of IFN-α.

### Blocking of NK cells elevates antibody response in Ad49L-nCoV-S immunized C57 mice.

**FIG 5:**
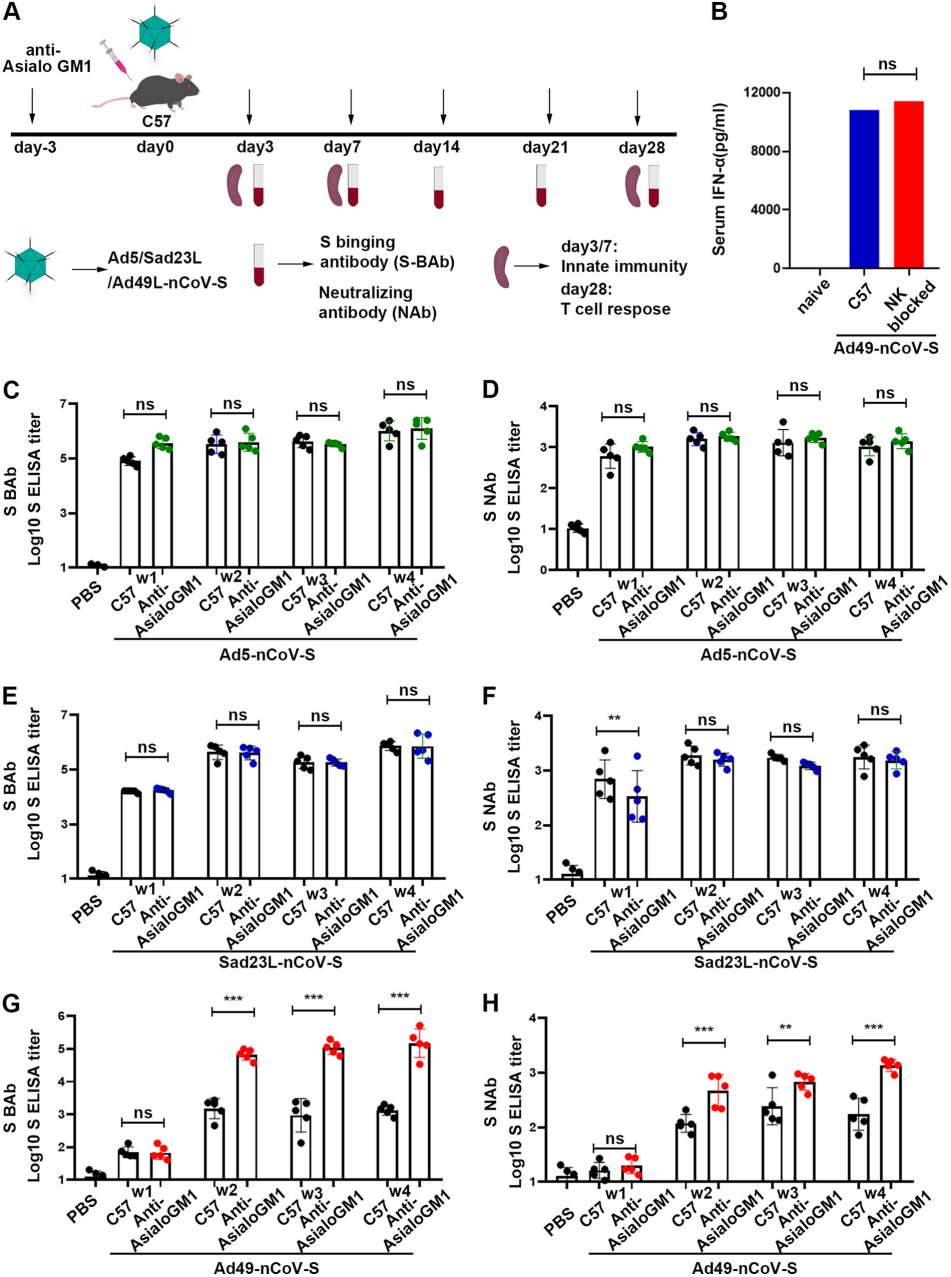
Specific antibody response of normal C57 and NK-blocked C57 mice immunized with Ad5-nCoV-S, Sad23L-nCoV-S, or Ad49L-nCoV-S. (A) Normal C57 and NK-blocked C57 mice (*n* = 5/group) were immunized by a dose of 10^9^ PFU Ad5-nCoV-S, Sad23L-nCoV-S, or Ad49L-nCoV-S. (B) Level of IFN-α in the serum of C57 and NK-blocked mice immunized with Ad49L-nCoV-S. (C, D) S-BAb and NAb titers of C57 and NK-blocked C57 mice immunized with Ad5-nCoV-S. (E, F) S-BAb and NAb titers of C57 and NK cells mice immunized with Sad23L-nCoV-S. (G, H) S-BAb and NAb titers of C57 and NK-blocked C57 mice immunized with Ad49L-nCoV-S. Statistically significant differences are shown with asterisks (*, *P* < 0.05; **, *P* < 0.01 and ***, *P* < 0.001).

**FIG 6:**
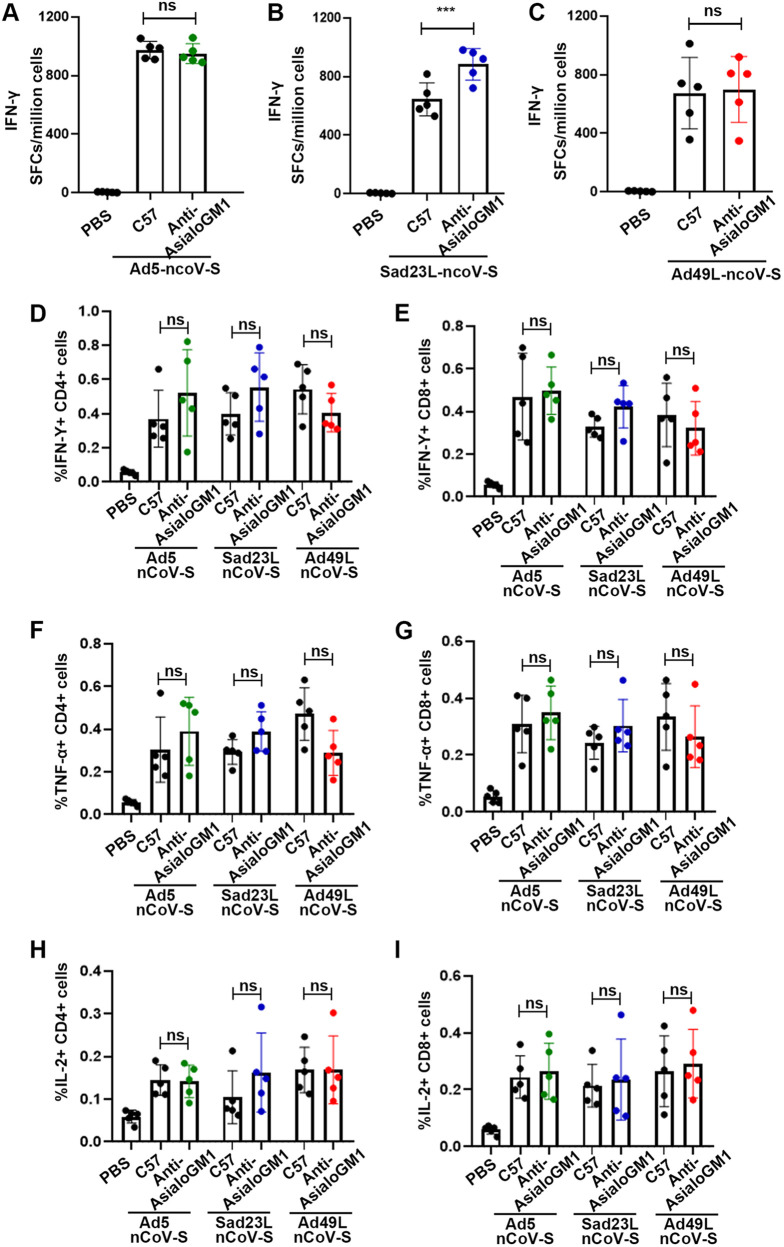
T cell response of normal C57 and NK-blocked C57 mice immunized with Ad5-nCoV-S, Sad23L-nCoV-S, or Ad49L-nCoV-S. Normal C57 and NK-blocked mice (*n* = 5/group) were immunized by a dose of 10^9^ PFU Ad5-nCoV-S, Sad23L-nCoV-S or Ad49L-nCoV-S. (A–C) IFN-γ secreting T-cell response of splenocytes to S peptides from Ad5-nCoV-S, Sad23L-nCoV-S, or Ad49L-nCoV-S immunized C57 and NK-blocked mice was measured by ELISpot. (D–I) Frequency of IFN-γ, TNF-α or IL-2 expressing CD4^+^ or CD8^+^ T-cell response to S peptides determined by ICS. Statistically significant differences are shown with asterisks (*, *P* < 0.05; **, *P* < 0.01 and ***, *P* < 0.001).

To investigate whether IFN-α downregulated specific antibody response was interrelated with NK cells, C57 mice were injected with 75 μg/day Anti-AsialoGM1 polyclonal antibodies for blocking NK cells for 3 days, and then the mice were immunized by Sad23L-nCoV-S, Ad49L-nCoV-S, or Ad5-nCoV-S vaccine (Fig. 5A). IFN-α level remained similar between normal and NK-blocked C57 mice immunized with Ad49L-nCoV-S (Fig. 5B) (*P* > 0.05). Interestingly, similarly high antigen-specific antibody responses were observed between normal and NK-blocked mice immunized with Sad23L-nCoV-S or Ad5-nCoV-S during 4 weeks postimmunization (*P* > 0.05; Fig. 5C-F), while Ad49L-nCov-S induced S-BAb and NAb responses in NK-blocked mice were higher than those in normal C57 mice (*P* < 0.001; Fig. 5G and H), but similar to Sad23L-nCoV-S in NK-blocked mice (Fig. 5E and F). The levels of antigen-specific T cell responses raised by these three adenovirus vectored vaccines had no significant difference mostly between normal and NK-blocked C57 mice (Fig. 6). These data indicated that blocking of NK cells could increase the level of S-BAb and NAb responses in Ad49L-nCoV-S vaccinated C57 mice even though IFN-α was at the high level.

### Blocking of NK cells increases specific CD4^+^ T_FH_ cells in Ad49L-nCoV-S immunized C57 mice.

**FIG 7:**
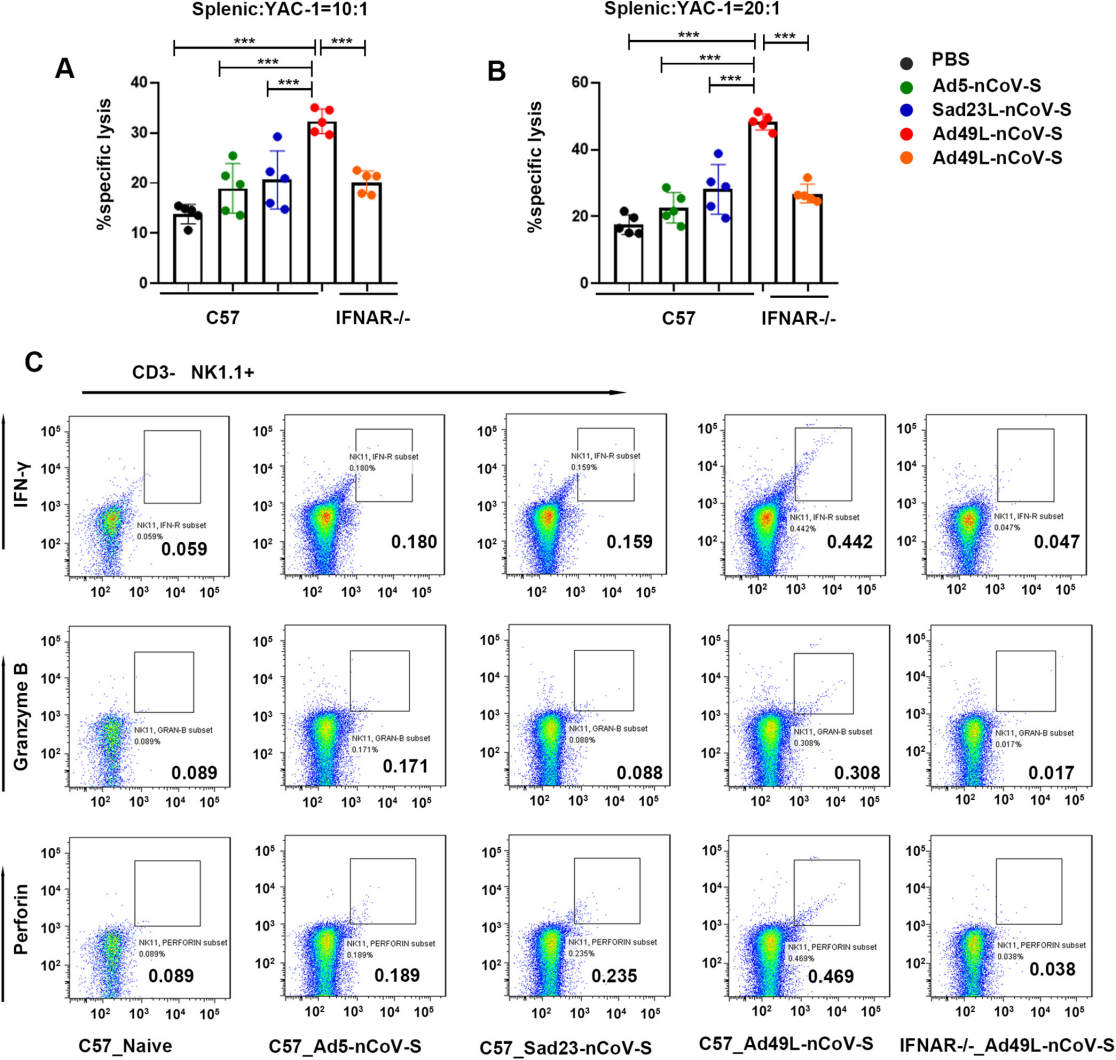
Function of activated NK cells in wild-type C57 and IFNAR^−/−^ C57 mice elicited with Ad5-nCoV-S, Sad23L-nCoV-S, or Ad49L-nCoV-S. Splenic cells of wild-type C57 and IFNAR^−/−^ C57 mice immunized with Ad5-nCoV-S, Sad23L-nCoV-S, or Ad49L-nCoV-S were measured for activation of NK cells by the cell cytotoxicity assay and ICS at day 3 after infection. (A, B) NK cell cytotoxicity of C57 and IFNAR^−/−^ mice immunized with Ad5-nCoV-S, Sad23L-nCoV-S or Ad49L-nCoV-S. (C) Splenic NK cells from C57 and IFNAR^−/−^ mice were stimulated for 4 h with polymethacrylic acid and ionomycin, and measured for intracellular IFN-γ, granzyme B and perforin production, respectively. Statistically significant differences are shown with asterisks (*, *P* < 0.05; **, *P* < 0.01 and ***, *P* < 0.001).

**FIG 8:**
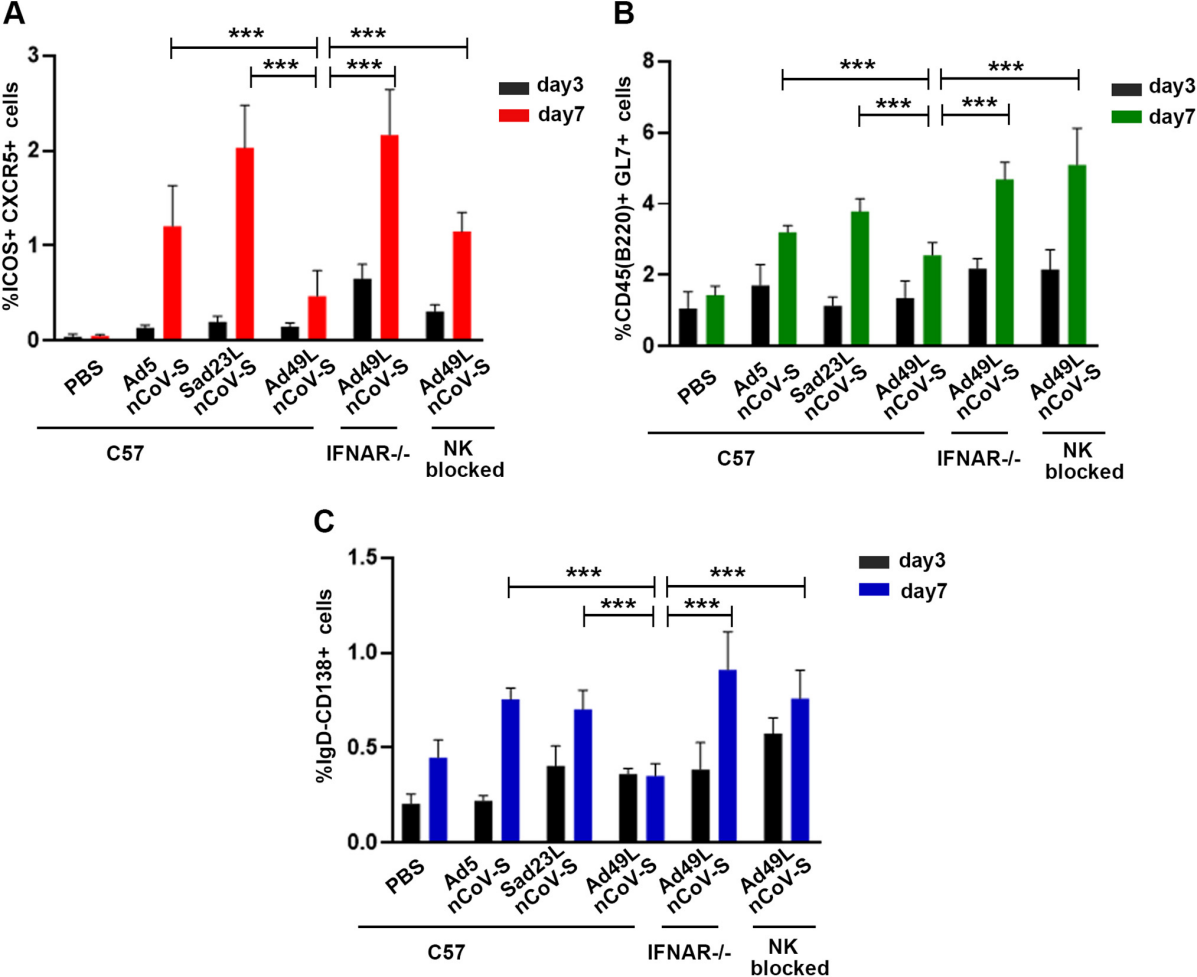
Activation of NK cells relying on early IFN-α activation in C57 mice immunized with different adenovirus vectored vaccines. Splenic cells of normal, IFNAR^−/−^, and NK-blocked C57 mice immunized with Ad5-nCoV-S, Sad23L-nCoV-S, or Ad49L-nCoV-S were measured for the number of CD4^+^ T_FH_, GC B, and ACS cells by ICS at days 3 and 7 after inoculation. (A–C) Frequency of T_FH_ cells, GC B cells and plasmacytes determined by ICS.

The activation of NK cells was examined by inducing with Sad23L-nCoV-S, Ad49L-nCoV-S, and Ad5-nCoV-S, respectively. Splenic NK cells of Ad49L-nCoV-S infected C57 mice were functionally activated by the evidence with the lytic activity of NK-sensitive YAC-1 cells (Fig. 7A and B) (*P* < 0.001). In contrast, splenic NK cells of IFNAR^−/−^ mice infected with Ad49L-nCoV-S were not activated, of which the percentage of specific lysis was similar to that of PBS control and Ad5 or Sad23L vectored vaccine (Fig. 7A and B) (*P* > 0.05). Ad49L-nCoV-S activated splenic NK cells of C57 mice produced significantly larger amounts of effector molecules such as IFN-γ, perforin, and granzyme B than those nonactivated splenic NK cells of normal and IFNAR^−/−^ C57 mice at day 3 post induction (*P* < 0.001; Fig. 7C). Furthermore, the number of CD4^+^ T_FH_ cells (ICOS^+^ CXCR5^+^) in Ad49L-nCoV-S infected C57 mice was significantly less than those in Ad49L-nCoV-S infected IFNAR^−/−^ and NK-blocked C57 mice, and Sad23L-nCoV-S or Ad5-nCoV-S infected C57 mice at day 7 postinfection (*P* < 0.001; Fig. 8A). Along with declining of CD4^+^T_FH_ cells in Ad49L-nCoV-S infected C57 mice, the numbers of GC B cells (B220^+^ GL7^+^) and plasma cells (IgD- CD138^+^) were decreased significantly (Fig. 8B and C) (*P* < 0.001). Interestingly, the numbers of CD4^+^ T_FH_ cells, GC B cells and plasma cells were restored in Ad49L-nCoV-S infected IFNAR^−/−^ mice and NK-blocked mice at day 7 postinfection (Fig. 8A to C).

To determine whether NK cells could directly affect B cells, 100 μg/day anti-CD4 monoclonal antibody and 75 μg/day Anti-AsialoGM1 polyclonal antibodies were intraperitoneally injected to Ad49L-nCoV-S immunized C57 mice to block T_FH_ and NK cells for 3 days. The data showed that S-BAb and NAb responses were not detected from both T_FH_ and NK blocked C57 mice in weeks 1 and 2 post injection but detected in weeks 3 and 4 closer to those from C57 mice (Fig. S1A and S1B), while the IFN-γ secreting T-cell response was lower in both T_FH_ and NK blocked C57 mice than that in C57 mice (Fig. S1C) (*P* < 0.001). The results suggested that the function of T_FH_ cells would determine B and T cell responses.

## DISCUSSION

In this study, the immune responses were extensively characterized from novel Sad23L and Ad49L vectored COVID-19 vaccines in comparison with Ad5 vectored vaccine in mice. Sads23L-nCoV-S and Ad5-nCoV-S induced the high antigen-specific antibody response, but Ad49L-nCoV-S induced the low specific antibody response, while all these three vectors mediated the high specific T cell responses and varied insignificantly (Fig. 1). This phenomenon was previously seen in chimpanzee adenovirus type 68 (CAd68), human adenovirus type 4 (Ad4), and human adenovirus type 35 (Ad35) vectors (7–9). The immune mechanism of this difference between Sad23L and Ad49L vectors was explored for relying on IFN-I signaling that the high level of IFN-α was induced by Ad49L-nCoV-S but not Sad23L-nCoV-S and Ad5-nCoV-S in mice (Fig. 2). Furthermore, the role of IFN-α restricting the antibody response was confirmed by comparing IFNAR^−/−^ and normal C57 mice after immunizing with Ad49L-nCoV-S, showing that the specific antibody response was significantly increased in IFNAR^−/−^ mice, similar to Sad23L-nCoV-S immunized C57 mice (Fig. 3).

IFN-I was demonstrated for reversely correlating to antiviral B cell response (18, 19). A previous study suggested that IFN-I induced by human adenovirus type 28 (Ad28) and Ad35 had multiple effects on T cell immunogenicity (20). There is evidence that NK cells regulated B cell responses; in some studies, NK cells inhibited B cells (21–23). Interestingly, in our study, when anti-AsialoGM1 polyclonal antibodies were used to block NK cells in C57 mice and then immunized with Ad49L-nCoV-S, the high antigen-specific antibody response was obtained even though a high level of IFN-α in serum (Fig. 5B, G, and H), which were similar to those in IFNAR^−/−^ mice immunized with Ad49L-nCoV-S (Fig. 3E and F), suggesting that the activated NK cells would inhibit specific antibody response. There is evidence showing NK cells restrict the total CD4^+^ T cell response (24–26), of which T_FH_ cells play an essential part in GC B cell formation and subsequent antibody production (27, 28). In this study, the mode of NK cells affecting the B cell response was further identified by blocking both NK and T_FH_ cells with specific antibodies, showing that Ad49L vector mediated antibody response was disappeared completely in the early 2 weeks of Ad49L-nCoV-S infection (Fig. S1), suggesting T_FH_ cells played an important role in antibody production.

A study described that IFN-I inhibited B cell response by a chimpanzee adenovirus vector decreasing transgene expression (9). Another study indicated that activation of NK cells was dependent on IFN-α produced by exposure to Ad28 or Ad35, but not to Ad5, in which IFN-α induced activation of NK cells leading to the increased monocyte apoptosis and subsequent loss of vector-insert expression (29). A similar study found that NK cells were activated and accumulated in the liver upon adenoviral infection *in vivo*, leading to the loss of adenoviral genome and transgene expression in the liver (30). However, these studies did not explain the mechanism of IFN-I affecting antigen-specific B cell response.

IFN-I affecting antigen-specific B cell response has been seen in other viruses as well. Delayed and weakened NAb response alongside with T cell exhaustion represents characteristic features of LCMV as well as of human immunodeficiency virus (HIV), hepatitis B virus (HBV), and hepatitis C virus (HCV) infections (31–34). Previous studies found that IFN-I suppressed LCMV-specific B cell responses by modulating CD8^+^ T cell differentiation and guiding inflammatory monocyte assembling at lymphonodus to prevent antiviral B cell responses (35, 36). Our data deduced that IFN-α activated NK cells and consequently decreased the numbers of CD4^+^ T_FH_ cells and restricted antigen-specific B cell response in Ad49L vectored vaccine immunized mice.

The mechanisms underlying the differential induction of IFN-I by various adenovirus vectors have not been well defined. Recombinant adenovirus vectors can be sensed by cell-surface or endosomal pattern recognition receptors (PRRs) such as the Toll-like receptors, which can trigger the downstream activation and transcription of antiviral genes, including nuclear factor-k-gene binding (NF-κB), mitogen-activated protein kinases (MAPK), and interferon-regulatory factors (IRFs) (37). One study indicated that IFN-α induction correlated with the permissibility of plasmacytoid dendritic cells (pDCs) to CD46, but not coxsackievirus and adenovirus receptor (CAR)-utilizing Ad serotypes (38, 39). And the viral DNA itself can play a crucial role in triggering innate immune responses. The engagement of cyclic GMP–AMP synthase (cGAS) triggers a signaling cascade involving the adaptor stimulator of interferon genes (STING) and activation of the TANK binding kinase 1 (TBK1), which initiate the induction of interferon regulatory factor 3 (IRF3)-responsive genes, such as IFN-I (40). The study showed that loss of the cGAS/STING pathway did not affect viral clearance, and cGAS deficiency had a modest influence on the magnitude of the antiviral humoral immune response to adenovirus infections (40). However, it did not compare with serotypes of adenovirus vectors. Further research is required to determine the key factors responsible for the differential induction of IFN-I by various adenovirus vectors.

In conclusion, besides raising an overall high cellular immunity, these two novel adenovirus vectors mediated differential specific antibody responses, of which Sad23L induced the high but Ad49L induced the low specific antibody activity. Ad49L-vectored vaccine initiated the high IFN-α and activated NK cells to inhibit antibody response via downregulating the number of CD4^+^ T_FH_ cells leading to the decline of GC B cells and plasma cells, and the subsequent neutralizing antibody production (Fig. S2). Sad23L or Ad49L vectored vaccine could be considered for prime-boost vaccination in combination or privileged for humoral or cellular immunization against a particular disease.

## MATERIALS AND METHODS

### Mice and adenoviruses.

Normal C57 mice as wild-type control were obtained from the Animal Experimental Centre of Southern Medical University, Guangdong, China. IFNAR^–/–^ defective C57 mice were purchased from the Institute of Laboratory Animals Science, CAMS & PUMC. All experiments were conducted in compliance with the guidelines for the care and use of laboratory animals and approved by the Southern Medical University (SMU) Animal Care and Use Committee at Nanfang Hospital, SMU, Guangzhou, China.

Recombinant E1- and E3-deleted Ad5, Ad49L, and Sad23L vectored COVID-19 vaccines (Ad5-nCoV-S, Ad49L-nCoV-S, Sad23L-nCoV-S) carrying the full-length gene of SARS-CoV-2 S protein were provided from the laboratory (11). Ad5-nCoV-S, Ad49L-nCoV-S, and Sad23L-nCoV-S were propagated from HEK-293A packaging cells and were purified by two rounds of CsCl density centrifugation as described previously (11).

### Antibody and flow cytometry.

Single-cell suspension was prepared from mouse spleen. Splenocytes were stained in combination with fluorescently labeled MAbs specific for CD45^+^(B220^+^), CD3, CD4, CD8, CD138, GL7, ICOS, CXCR5, IgD, NK1.1, and Thy1.1. Anti-AsialoGM1 polyclonal antibodies were used to block NK cells. All antibodies were purchased from eBioscience (San Diego, CA, USA). For intracellular cytokine staining, mouse splenocytes (2 × 10^6^ cells/well) were stimulated with S peptide pool (3 μg/mL each peptide) or negative control medium in triplicates. After 4 h, the cells were incubated with Golgi Plug (BD Bioscience, NJ, USA) for 6 h at 37°C. Cells were collected and stained with anti-mouse CD3, CD4 and CD8 surface marker antibodies (eBioscience, San Diego, CA, USA). Cells were fixed with IC fixation buffer, permeabilized with permeabilization buffer (BD Bioscience, NJ, USA), and stained with anti-mouse IFN-γ, IL-2, and TNF-α (eBioscience, San Diego, CA, USA). All samples were tested with BD FACS Canton flow cytometer (BD Bioscience, NJ, USA).

### *In vitro* dendritic cell culture and IFN-α measurement.

DCs were generated from bone marrow of 4- to 6-week-old male C57 mice. Briefly, bone marrow cells were harvested from the femurs and tibiae of mice and cultured in the presence of murine GM-CSF (20 ng/mL) and IL-4 (10 ng/mL) (PeproTech, USA). On day 10, DCs were stimulated with 10^9^ PFU Ad5-nCoV-S, Ad49L-nCoV-S or Sad23L-nCoV-S for 24 h, in which the culture supernatants were measured for IFN-α by QuantiCyto Mouse IFN-α ELISA kit (Neobioscience, China).

### ELISA.

The microtiter plates (Corning, USA) were coated overnight with 2 μg/mL SARS-CoV-2 S proteins (Sino Biological, China). Serum samples were 2-fold serially diluted and S-BAb was detected by ELISA. Secondary antibodies were goat anti-mouse IgG-HRP (Beijing Bersee Science and Technology, Co. Ltd., China). Endpoint titers were defined as the highest reciprocal serum dilution that yielded an absorbance >0.2 and a ratio of signal than cutoff (S/CO) >1. Log_10_ endpoint titers were reported.

### pVNT.

Pseudovirus expressing a luciferase reporter gene and SARS-CoV-2 S protein gene was generated for measuring of NAb to SARS-CoV-2 as previously described (12). Briefly, pseudovirus was obtain from HEK-293 T cells cotransfected with plasmid psPAX2 (Addgene), pLenti-CMV Puro-Luc (Addgene), and pcDNA3.1-SARS-CoV-2 S. The pVNT titer (50% inhibitory concentration, IC_50_) was measured with HEK293T-hACE2 cells. Threefold serial dilutions of heat-inactivated vaccinated serum samples were prepared and mixed with 50 μL of pseudovirus for 1h. The serum-virus mixture was added to 5 × 10^4^ HEK293T-hACE2 cells. After 48 h of incubation, cells were measured by Steady-Glo Luciferase Assay (Promega) according to the manufacturer’s instructions. The pVNT IC_50_ titer of SARS-CoV-2 antibody was defined as the sample dilution at which a 50% inhibition rate. Inhibition rate (%) = (1 − sample RLU/virus control RLU) × 100. Log_10_ pVNT (IC_50_) titer was reported.

### ELISpot.

Mouse IFN-gamma ELISpot PLUS kits (MabTech) were used to determine SARS-CoV-2 S antigen-specific T lymphocyte response. Mouse splenocytes (5 × 10^5^ cells/well) were stimulated with S peptides (5 μg/mL) in triplicates. Fifty-four peptides encoding amino acid sequence of SARS-CoV-2 S protein were predicted (http://www.iedb.org/) and synthesized by Guangzhou IGE Biotechnology LTD (11). Spots were counted with a CTL Immunospot Reader (Cellular Technology Ltd.). The results were expressed as spot forming cells (SFCs) per million cells.

### NK cell cytotoxicity assay.

NK cell cytotoxicity was detected by CyQUANT LDH cytotoxicity kit (eBioscience, San Diego, CA, USA). Briefly, single-cell suspension of mouse splenocytes were then incubated with YAC-1 cells (1 × 10^4^ cells/well) at different effector-to-target cell ratios for 2 h at 37°C, and then 50 μL of each sample culture were transferred to a 96-well flat-bottom plate in triplicate to mix with 50 μL of the substrate for 30 min. Finally, 50 μL of the stop solution were added to each sample well. The absorbance at 490 nm and 680 nm was read, respectively, and the cytotoxicity (%) was calculated by using the following formula:

%cytotoxicity=(compound–treated LDH activity−spontaneous LDH activity)/(maximum LDH activity−spontaneous LDH)×100.

### Statistical analyses.

S-BAb and NAb titers between different groups were compared using two-tailed *t* test and one-way ANOVA. IFN-γ secreting T cell SFCSs, cytokine rates, YAC-1 cell lysis rates and IFN-α levels among groups were compared by two-tailed *t* test, one-way ANOVA and Mann–Whitney test. The graphs were conducted by GraphPad Prism version 8 (GraphPad Software, La Jolla, CA, USA). All the statistical analyses were computed with SPSS version 21.0 (SPSS Inc., Chicago, IL, USA). Statistically significant differences are shown with asterisks (*, *P* < 0.05; **, *P* < 0.01 and ***, *P* < 0.001).
