# Supplementary material for: Two Novel Adenovirus Vectors Mediated Differential Antibody Responses via Interferon-α and Natural Killer Cells
Source: Microbiol Spectr. 2023 Jun 22;11(4):e00880-23. doi: 10.1128/spectrum.00880-23 (PMC10434031; doi:10.1128/spectrum.00880-23)
Supplement: Supplemental file 1 — Fig. S1 and S2. Download spectrum.00880-23-s0001.pdf, PDF file, 0.2 MB [file spectrum.00880-23-s0001.pdf]

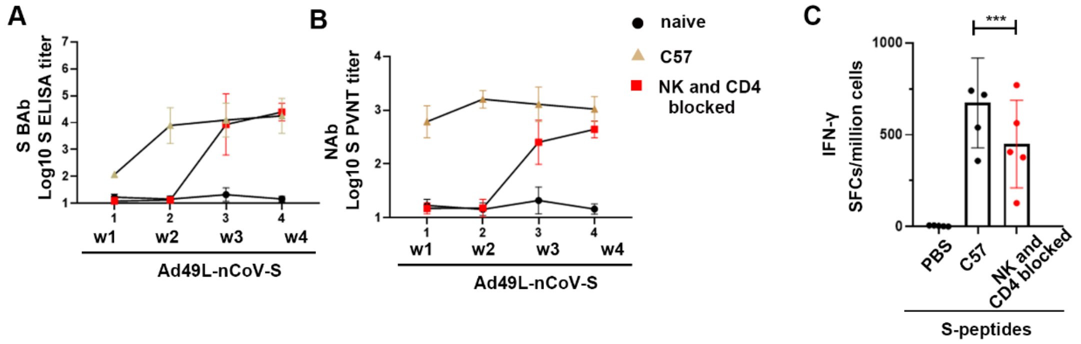

Fig. S1

Specific antibody and T cell responses from normal and both NK and CD4 blocked C57 mice immunized with Ad49L-nCoV-S. (A, B) S-BAb and NAb titers from C57, and both NK and CD4 blocked C57 mice immunized with Ad49-nCoV-S. (C) IFN-γ secreting T-cell response (spot forming cells [SFCs]/million cells) of splenocytes to S peptides from Ad49L-nCoV-S immunized mice was measured by ELISpot.

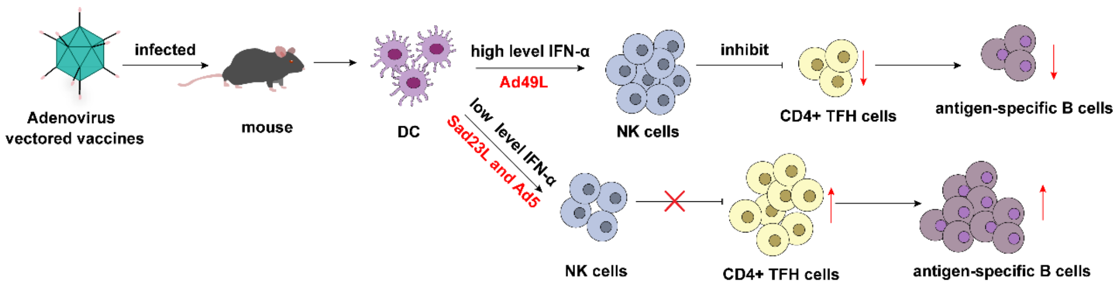

Fig. S2

The proposed mechanism for IFN-α differentiating antigen-specific antibody responses in Sad23L or Ad49L vectored vaccine immunized mice.
